# VISTA: VIsual Semantic Tissue Analysis for pancreatic disease quantification in murine cohorts

**DOI:** 10.1038/s41598-020-78061-3

**Published:** 2020-12-01

**Authors:** Luke Ternes, Ge Huang, Christian Lanciault, Guillaume Thibault, Rachelle Riggers, Joe W. Gray, John Muschler, Young Hwan Chang

**Affiliations:** 1 Department of Biomedical Engineering and OHSU Center for Spatial Systems Biomedicine (OCSSB), Portland, OR USA; 2 Computational Biology Program, Oregon Health & Science University, Portland, OR USA; 3 Department of Pathology, Oregon Health & Science University, Portland, OR USA; 4 Knight Cancer Institute, Portland, OR USA; 5 Brenden-Colson Center for Pancreatic Care, Portland, OR USA

**Keywords:** Software, Cancer, Biomedical engineering, Computational science, Computer science, Image processing, Software, Machine learning

## Abstract

Mechanistic disease progression studies using animal models require objective and quantifiable assessment of tissue pathology. Currently quantification relies heavily on staining methods which can be expensive, labor/time-intensive, inconsistent across laboratories and batch, and produce uneven staining that is prone to misinterpretation and investigator bias. We developed an automated semantic segmentation tool utilizing deep learning for rapid and objective quantification of histologic features relying solely on hematoxylin and eosin stained pancreatic tissue sections. The tool segments normal acinar structures, the ductal phenotype of acinar-to-ductal metaplasia (ADM), and dysplasia with Dice coefficients of 0.79, 0.70, and 0.79, respectively. To deal with inaccurate pixelwise manual annotations, prediction accuracy was also evaluated against biological truth using immunostaining mean structural similarity indexes (SSIM) of 0.925 and 0.920 for amylase and pan-keratin respectively. Our tool’s disease area quantifications were correlated to the quantifications of immunostaining markers (DAPI, amylase, and cytokeratins; Spearman correlation score = 0.86, 0.97, and 0.92) in unseen dataset (n = 25). Moreover, our tool distinguishes ADM from dysplasia, which are not reliably distinguished with immunostaining, and demonstrates generalizability across murine cohorts with pancreatic disease. We quantified the changes in histologic feature abundance for murine cohorts with oncogenic Kras-driven disease, and the predictions fit biological expectations, showing stromal expansion, a reduction of normal acinar tissue, and an increase in both ADM and dysplasia as disease progresses. Our tool promises to accelerate and improve the quantification of pancreatic disease in animal studies and become a unifying quantification tool across laboratories.

## Introduction

Advances in deep learning technologies are creating opportunities for the rapid and objective assessment of both normal tissue and pathologic processes in biologic specimens. Computer-aided interrogation of medical imaging is being applied to accelerate and improve diagnosis in human patients^1–4^. Similarly, deep learning technologies can greatly improve analyses in animal disease models which require the measurement of disease progression in large numbers of tissue samples resulting either from pharmacological or genetic manipulations. The extensive and growing use of murine models in disease studies creates a significant need for tissue assessment methods that are rapid, objective and quantifiable in order to permit statistically validated disease measurements among animal cohorts, free of technical variability and investigator bias.

The challenge of objective quantification of tissue changes among animal cohorts is significant. Evaluation of tissue by either histochemical stains or antigen-specific immunohistochemistry offers distinct and sometimes overlapping information, but both have limitations. Hematoxylin and eosin (H&E) staining is a rapid, reliable and inexpensive method; however, lack of molecular specificity and requirement for manual segmentation have, thus far, limited its use for extraction of quantifiable data. Consequently, disease assessments by H&E staining are typically qualitative and vulnerable to inter-observer variation and bias^5–7^. Immunohistochemical stains offer a degree of specificity, but immunostaining can be labor- and time-intensive, expensive and results are often challenging to objectively quantify over broad tissue regions. In addition, tissue features of interest are not always cleanly distinguishable by immunostaining markers, and so tissue assessments can be limited by reliance on the molecular specificity of antibodies.

Using murine models of pancreatic cancer progression and pancreatitis, we are working to develop and validate deep learning approaches that enable the rapid, reliable, and automated quantification of disease progression over large tissue areas, solely based on H&E staining. Murine models of pancreatic cancer were chosen as they have proven useful for mechanistic investigations of pancreatic cancer progression, modeling well the human disease both genetically and phenotypically, particularly during the evolution of pre-cancerous lesions^8,9^. The murine models have produced an explosion of studies including pre-clinical drug tests and evaluation of additional genetic perturbations that expose tumor-suppressing and tumor-promoting disease modifiers^10–12^.

The early stages of pancreatic cancer evolution are well described in the mouse models^8,9^. The normal pancreas consists predominantly of acinar and ductal epithelial cells forming the exocrine compartment, along with islet cells of the endocrine compartment, vasculature and the varied fibroblasts of the stromal compartment. The earliest stages of oncogene-induced pre-cancer evolution are marked by an expansion of ductal cells or by the conversion of the acinar cells to a ductal phenotype in an adaptive process known as acinar-to ductal metaplasia (ADM)^13^. ADM is also characteristic of acute and chronic pancreatitis, inflammatory conditions that can predispose to cancer^13^. The next stage in cancer evolution is the development of low-grade dysplasia, also referred to as pancreatic intraepithelial neoplasias (PanINs 1 and 2). Low-grade dysplasia is a pre-invasive neoplasia that can evolve to high-grade dysplasia (PanIN 3) and then progress to invasive pancreatic ductal adenocarcinoma (PDAC)^14^. Both ADM and dysplasia are accompanied by a prominent stromal reaction and immune cell infiltrate^13^. The stages of ADM and dysplasia evolution are believed to encompass a long phase of pre-cancer evolution that is a valuable window for early intervention^14^.

Here we describe the model training workflow and application of deep learning on H&E stained samples of murine pre-cancerous lesions, segmenting the normal acini, the ductal phenotype of ADM, and dysplasia. With the rapid growth of computer vision, more specifically deep learning, novel image analysis architectures have been developed for accessing image information that is not readily observed through traditional methods. Several research groups have worked towards inter-modality image translation and have developed tools that attempt to convert medical images such as H&E stained tissue and brightfield microscopy to more detailed ones such as fluorescent immunostains^15–18^. The target of such models has been the direct translation of stain intensities for the purpose of constructing entirely new images. Our developed tool seeks to go further, predicting binarized masks of positive staining area and augment immunostaining by segmenting key histologic features that current stains cannot reliably differentiate.

Results presented here demonstrate a well validated segmentation tool that can automatically, rapidly, and objectively quantify pancreatic tissue and disease progression in mice, relying solely on easily replicated and low-cost H&E staining of whole pancreas tissue sections, free of experimental variability and investigator bias. Our work provides a tool that is immediately applicable to the improvement and acceleration of pancreatic disease studies in animal cohorts, and provides workflows for similar tool development in other disease models. Moreover, the ease of use and availability allows for this tool to be a common thread for comparing different studies performed throughout the world.

## Results

In order to predict the histologic feature distributions and immunofluorescent stain positivity in murine pancreatic pre-cancerous tissues, several UNet models^19^ were trained using intensity normalized H&E image tiles paired with annotated ground truth tiles (Supplemental Fig. 1). All pancreas tissue sections in training, validation, and testing sets were stained with H&E (Table 1). First testing was conducted by evaluating spatial overlap of predictions and expert annotations for normal acinar, ADM, and dysplasia. A second test was performed by correlating predictions to binarized immunofluorescence staining (IF): amylase (AMY), labeling normal acini, pan-keratin (panK), labeling primarily the oncogenic Kras-transformed epithelial population, and DAPI, labeling all nuclei. A third test was performed qualitatively analyzing predictions in pancreatitis and normal samples, and comparing to biological expectations.

**Table 1:** Datasets used.

	Sample size	H&E	IF	Annotations	Used for
KC (2 months)	12	x	x		IF correlation Area evaluations in pre-cancer histopathology
KC (5 months)	16	x	x (n = 13)	x (n = 3)	Training and validation Dice evaluation (H&E based prediction vs annotations) IF Spearman correlations Area evaluations in pre-cancer histopathology SSIM evaluation (H&E based prediction vs IF stain) Synthetic stain generalizability
Induced pancreatitis	6	x			New tissue generalizability
Normal tissue	3	x			New tissue generalizability

To ensure that UNet^19^ model predictions were able to generalize well and overcome staining differences within and between tissue sections, Dice Coefficient were calculated comparing model predictions to expert annotations made in Cytomine^20^ after training with several different normalization techniques (Reinhard^21^, Vahadane^22^, and Macenko^23^). As observed in Table 2, the models implementing Reinhard normalization achieved better scores on average, relative to Vahadane and Macenko. Furthermore, the models achieved the best scores when the normalization process was applied on intermediate crops rather than across the whole image. This is because staining can be uneven within a single section, and normalizing crops helps to overcome these differences in intensity; whereas, normalizing across a whole section only helps overcome differences between images.

**Table 2:** Evaluation of model performances.

Normalization method	Metric	Normal acinar	ADM	Dysplasia
Reinhard normalization of intermediate crops	Dice	**0.78691**	**0.70239**	**0.79403**
	BCE	0.16131	0.17112	0.22374
Reinhard normalization^21^	Dice	0.71750	0.60303	0.76210
	BCE	0.20561	0.16635	0.21966
Vahadane normalization^22^	Dice	0.69311	0.58241	0.73684
	BCE	0.20753	0.18726	0.24471
Macenko normalization^23^	Dice	0.70686	0.56660	0.77210
	BCE	0.21784	0.18370	0.19711

Bold shows best performance result.

**Figure 1:**
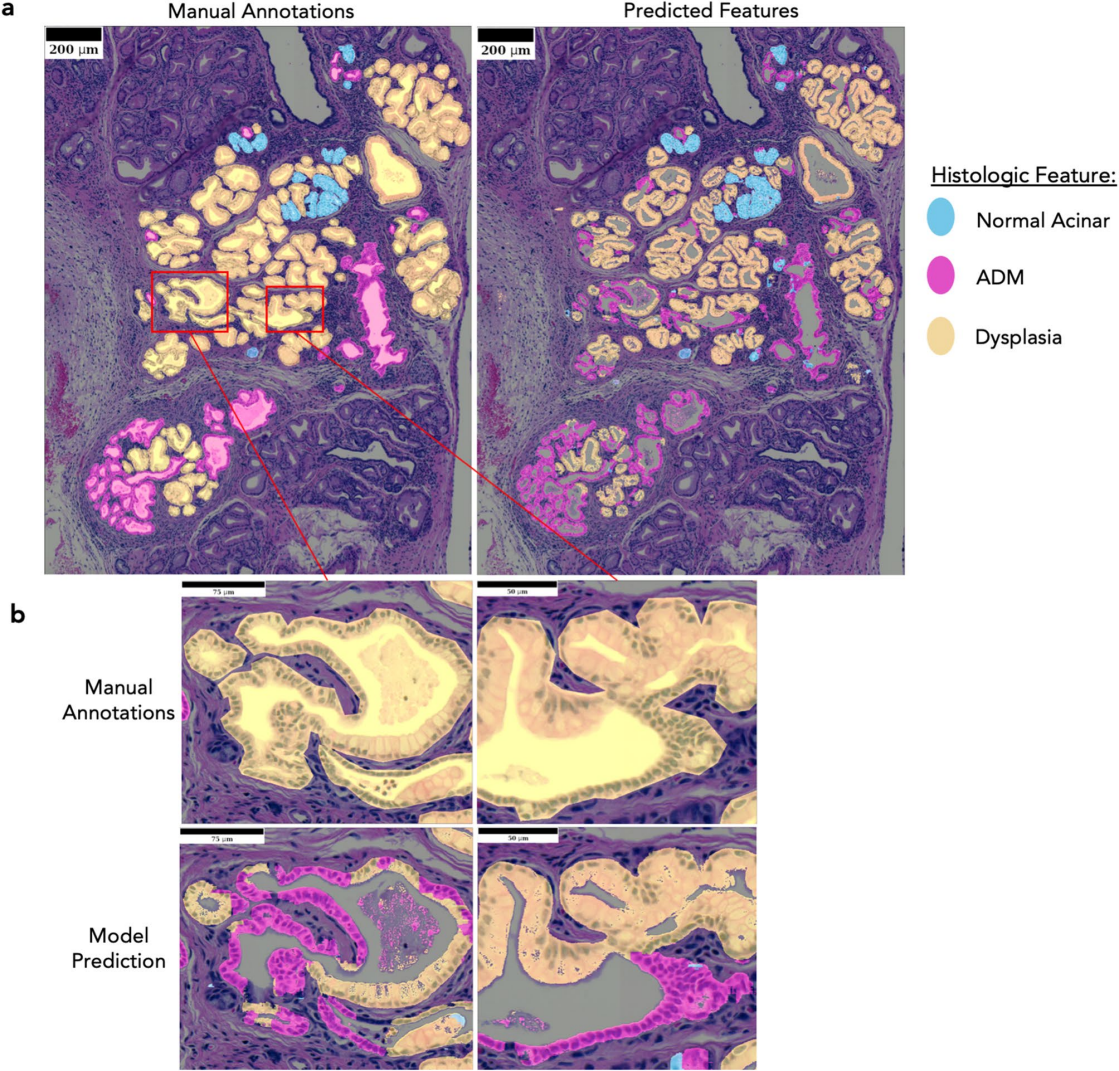
Predictions compared to annotations. (**a**) Model Predictions closely align with the manually annotated ground truth regions that was used for training. (**b**) Close inspection of the ducts shows consistent discrepancies regarding the lumen and split histologic features within single ducts. Manual annotations were made by circling whole ducts, but the models’ predictions are actually more reflective of biology, wherein, stain does not mark for the lumen. The Predictions can also distinguish histologic features differences that the manual annotations combined.

The models were trained using 80% of the training dataset, and 20% of that training dataset was held out for cross-validation to evaluate and tune the models’ performance with unbiased data. The unseen labeled test data, comprising 20% of the annotated dataset was used for final evaluation of the models. The best models yielded Dice Coefficients of ~ 0.79, ~ 0.70, and ~ 0.79 on the hold-out set for normal acinar tissue, ADM, and dysplastic features, respectively (Table 2). The segmentations match the expert annotations with a high degree of qualitative accuracy (Fig. 1a). The reason that the models’ Dice scores are lower than expected from successful models is because the models actually refined approximations in the experts’ annotations leading to discrepancies between prediction and annotation (Fig. 1b). Due to the limitations of the annotation method used, entire lesions (including empty lumina, mixed morphologies (Supplemental Fig. 2E), and additional negative space) were labeled as one type of tissue (i.e., ADM or dysplasia). The models, however, accurately differentiate between the tissue types within a lesion and avoid labeling lumina. Despite these results being biologically correct, they are different than the experts’ manual annotations, resulting in a negative impact on the measured Dice Coefficients.

**Figure 2:**
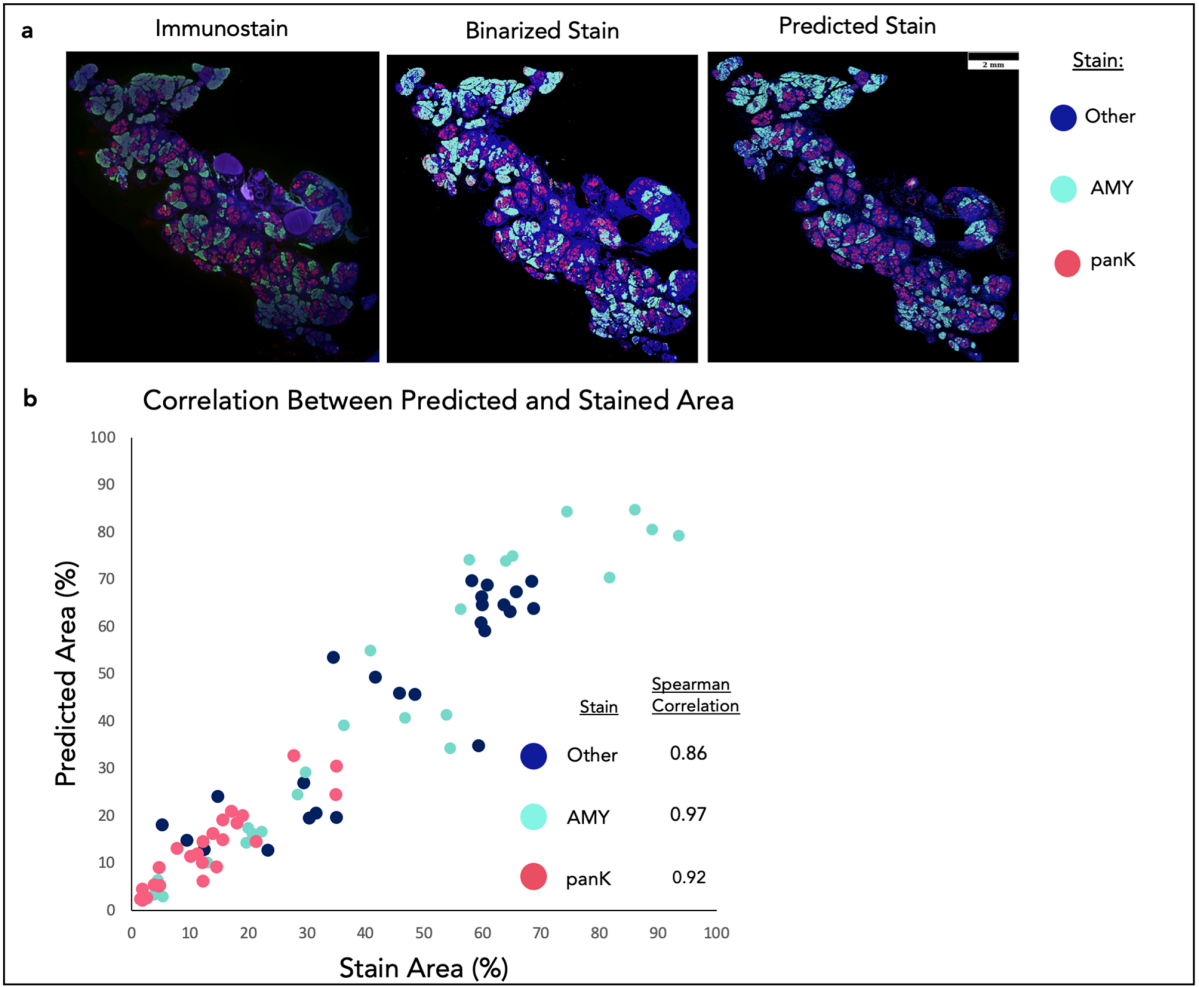
Comparing model predictions to stained tissue. (**a**) Stain masks and predicted segmentation masks are qualitatively highly similar. Differences can be seen in the high-level architecture of the tissues, which is indicative of the fact that the predictions were made from serial sections to the stains. There are also dim regions of the stained image that are lost from the global thresholding technique. These regions are successfully captured by the models. "Other" stain is the DAPI stain minus regions overlapping with AMY and panK. (**b**) Correlations were made by comparing the percent of area coverage for each stain mask. The high Spearman correlations illustrate the models’ ability to replicate straining using only H&E images. These regions are successfully captured by the models. "Other" stain is the DAPI stain minus regions overlapping with AMY and panK.

To test the accuracy of the trained models further, a comparison was made between quantified model predictions and a second unseen test set of immunostained images that have been binarized. Quantification of the tissue area occupied by normal acinar cell and transformed pancreatic epithelial cells was achieved by immunostaining for amylase and pan-keratin, respectively, with DAPI staining of nuclei used to detect all cellular regions. The comparable calculation was then made using tool predictions on adjacent H&E stained tissue sections. For the tool prediction, ADM and dysplasia predictions were grouped into the panK stain because pan-keratin immunostaining does not distinguish ADM and neoplastic tissues. Because stain area is specific and more biologically targeted than the rough annotations that incorporate empty lumens and mislabeled features, the models’ immunostain Spearman correlation scores are much more reflective of their overall accuracy and sensitivity. When the prediction masks are compared qualitatively and quantitatively to the stained images, the models are able to predict the spatial localization of the immunostaining (Fig. 2a and Supplemental Fig. 3). Prediction accuracy was evaluated against biological truth using immunostaining and structural similarity (SSIM) (Supplemental Fig. 3), in addition to the area correlations (Fig. 2). SSIM was chosen as our metric to evaluate against because it would be more robust than Dice against differences between serial sections. Note that H&E and IF stained samples were acquired from adjacent serial sections.

There are minor differences between the immunostained and the predicted segmentations, which reflects slight tissue variations between the adjacent, but separate, sections used for H&E and IF staining. Quantitatively, three models also have high Spearman correlations (Fig. 2b) with the immunostained sections despite these sections (n = 25) being unseen during training, with Spearman correlation values of 0.97, 0.92, and 0.86 for AMY, panK, and DAPI stained other tissue, respectively. These correlations are very strong, despite the assumption that the serial section have true correlation values of close to 1^24^. The good qualitative spatial localization and strong correlations validate that the models have been successfully trained and are capable of replicating known biological data.

**Figure 3:**
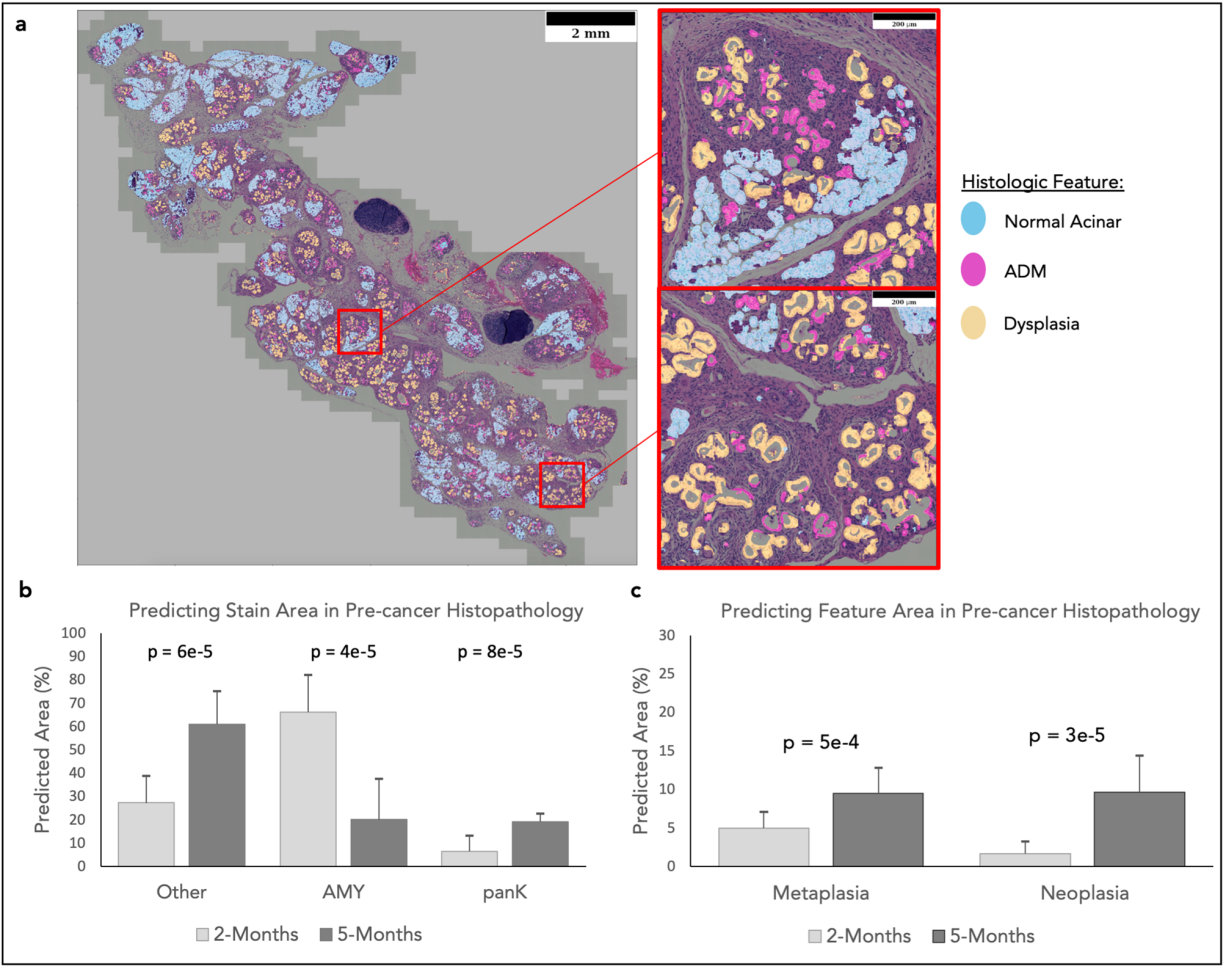
Discerning features beyond immunostaining. (**a**) In test images the predicted histologic features visually align with what is expected from the H&E images. This shows the models’ utility in discerning novel information regarding ductal features that cannot be detected via staining. The models were used to predict the changes stain distributions (**b**) and cancer histologic features (**c**) in murine models with induced cancer. Predictions show significant changes in all stains and features between time points, and quantifies specific features that were not discernable in immunostaining alone. Mann–Whitney U test was used to test for statistical analyses.

Not only can these models replicate immunostaining data, they can extract more information than can be gained via immunostaining. In the second unseen testing dataset consisting of 25 IF/H&E image pairs, the pan-keratin immunostain labels both metaplasia and dysplasia, restricting the disease features that can be segmented. The model predictions, however, can distinguish these features (Fig. 3a). This allows for deeper and more nuanced quantification of disease progression than can be achieved by immunostaining alone. Across a whole section of unseen test tissue, it can be observed that each predicted feature corresponds with the correct morphology.

**Figure 4:**
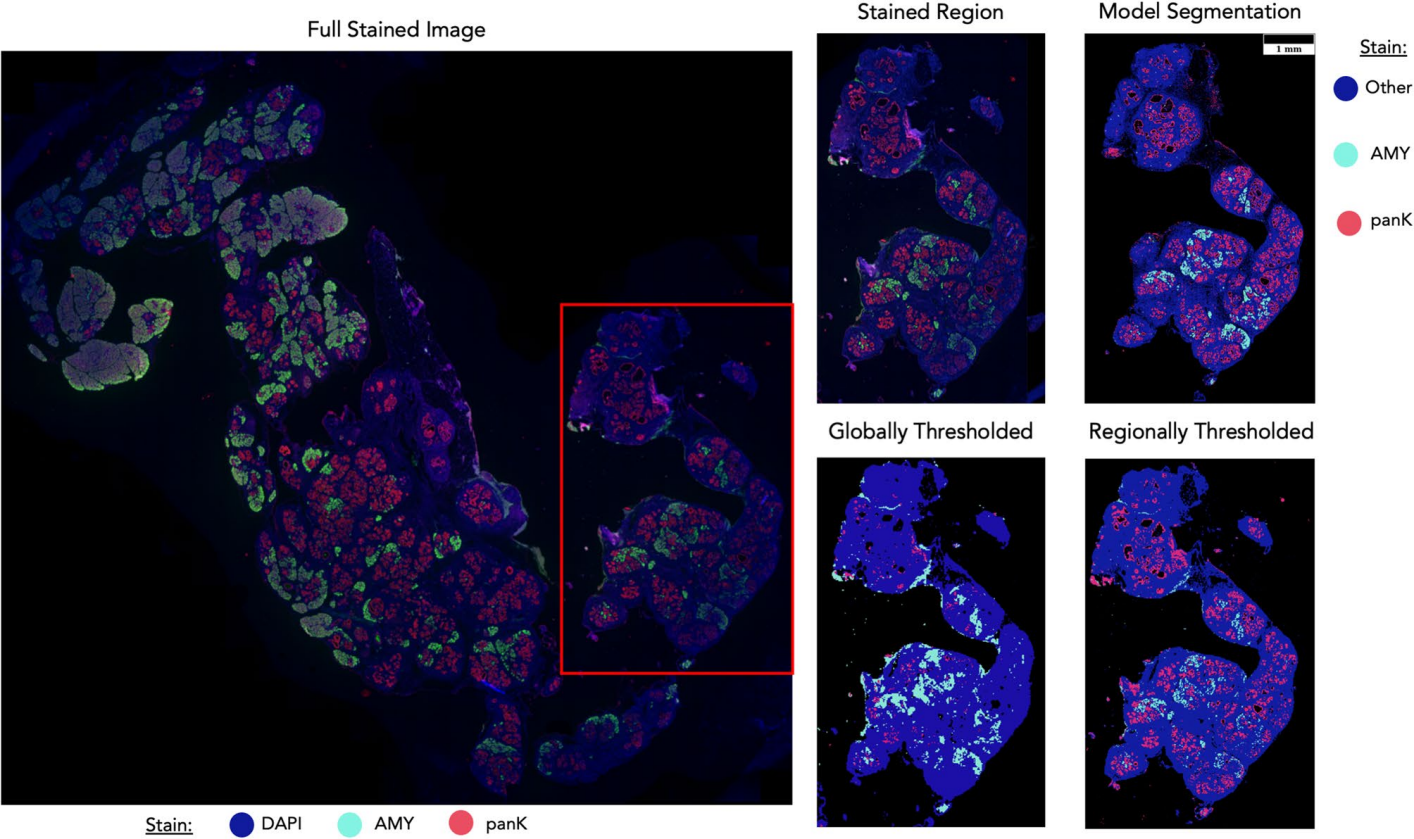
The problems with manual thresholding. The quality of the full stained image varies region to region, as some regions have dimmer staining than others. Because of this uneven staining quality, a single global threshold will not accurately represent true positives and negatives because dimmer regions will be neglected. When regions are thresholded independently, the quality of the segmentation masks improves; however, even regional dim spots are still dropped from the segmentations. The developed models, however, are able to overcome this limitation because it utilizes H&E images and is able to analyze the histologic features beyond just the intensity of the stain. “Other” stain is the DAPI stain minus regions overlapping with AMY and panK.

Because this process of prediction is deterministic, it is also a faster and less biased than manually annotating histologic features, and less expensive and less error-prone than immunostaining (Fig. 4). Standard binarization of whole slide IF stains often leaves dimmer regions of the tissue with inaccurate predictions of stain positivity. This process of setting a threshold for stain binarization is also a subjective process that will have different results depending on the expert looking at the image, and performing regional thresholding throughout the image demands more time and introduces more thresholds that can be biased by the evaluator. By comparison, the trained models are deterministic and are able to overcome staining differences in a consistent manner. Furthermore, the process of staining an IF section takes two days following standard protocol, with additional time spent image processing and binarizing the image afterwards. By comparison, the deep learning models take less than an hour depending on section size and graphics process unit (GPU) performance. Human annotation of the data is even slower, taking days to weeks for a single section and can have high variability between annotators. In addition, it can be difficult to get access to an expert with pathology certification necessary for differentiating the morphologies.

Using the tissue sections from the second unseen testing dataset isolated from *P48*^+*/Cre*^*; LSL-KRAS*^*G12D*^ (KC) mice at 2 and 5 months of age (n = 12, n = 13), the model was able to quantify tissue changes reflecting disease progression by predicting immunostain from H&E stain images (Fig. 3b,c). The observed age-dependent transitions from normal acinar to ADM and dysplasia, and the increase in other tissue area (DAPI stained), is consistent with biological expectations, illustrating the practical, objective use of this tool to quantitatively assess pre-cancerous disease development.

**Figure 5:**
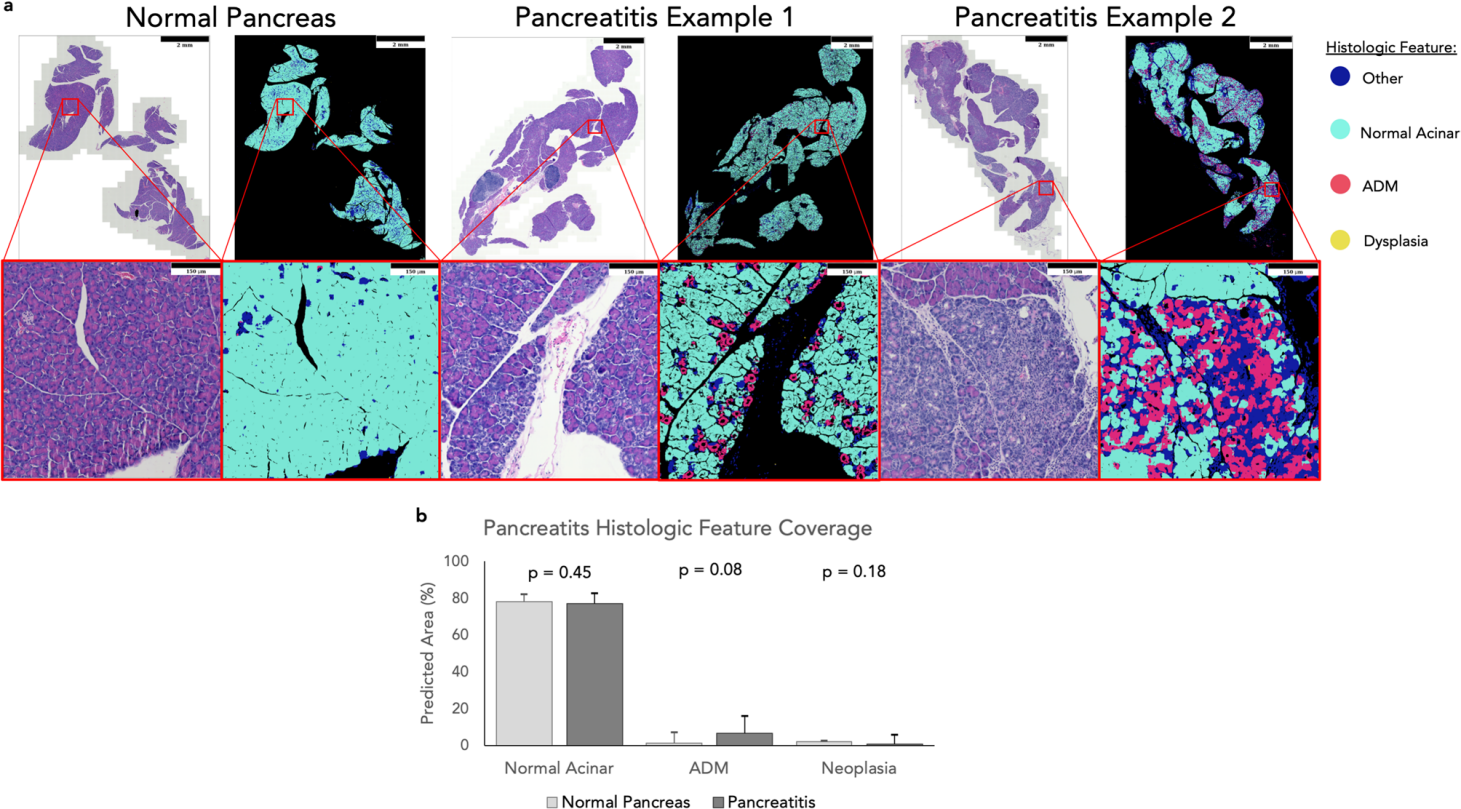
Predicting histologic features in pancreatitis. The model predicted histologic features match what in expected in both normal and pancreatitis samples. (**a**) Predicted images show that tissue is dominated by normal acinar with pockets of clear ADM localization. In normal tissue ADM and dysplasia are sparse predictions comprised primarily of arbitrary single pixels, and in pancreatitis this is true for just dysplasia. (**b**) In normal tissues, ADM and dysplasia predictions are negligible, and in pancreatitis there is a significant spike in ADM coverage with negligible dysplasia. Mann–Whitney U test was used to test for statistical analyses. Erroneous predictions of ADM and dysplasia in these samples are primarily driven by noise.

To test the models’ robustness and generalizability, we evaluated images from pancreata exhibiting histopathology associated with acute pancreatitis instead of histopathology induced uniquely by oncogenesis. Acute pancreatitis is characterized by prominent ADM and an inflammatory stromal response, but does not promote neoplastic lesions^13^. Acute pancreatitis was induced in mice by injection of the pro-inflammatory agent caerulein^13^, then tissue sections exhibiting acute pancreatitis or normal pancreas (n = 6, n = 3 respectively) were analyzed by the model (Fig. 5). This was performed on a third test dataset not seen by the models during training. Because neither annotations nor stains exist for this third dataset, model prediction localizations were evaluated qualitatively. Despite not being trained to analyze the particular disease states of pancreatitis, the models were able to accurately label pancreatitis features (i.e. ADM) with minimal error, regardless of whether the ADM was sporadic or clustered within the tissue (Fig. 5a). The model’s quantified tissue assessments show the significant presence of ADM by pixel area in the pancreatitis samples compared to normal tissues, which matches biological expectations. The near-absence of significant ADM and dysplasia in normal pancreas samples is also consistent with expectations, as is the near-absence of dysplasia in the pancreatitis samples (Fig. 5b). The small quantities of ADM and dysplasia predictions in the normal tissues are errors introduced primarily by pixel level noise and are insignificant compared to the size of the samples. Within this dataset we do not see large heterogeneity in the histologic features across disease states, and as a result the model performs consistently across all disease states shown.

## Discussion

The computational tool developed here is intended to augment and accelerate disease research performed in animal models by allowing for simple stain prediction and histologic feature labeling from H&E images without the need for expensive and time-consuming immunostaining and biased image interpretation. It can be used to both mark the localization of tissue features and quantitatively to measure the extent of disease based on multiple histologic features (Supplemental Fig. 2). Such rapid and unbiased quantification of disease states in animal models is critical to enabling efficient and accurate disease assessments among large study cohorts, as well as provide a common method to compare finding across different studies. The ability of this tool to accurately predict histologic features among 25 unseen pancreatic pre-cancer samples from multiple time points and 9 unseen samples comprising other disease states demonstrates the robustness of the models when analyzing new datasets. The fact that the models generalize well, despite being trained with a relatively small dataset (Supplemental Fig. 4 and Table 3), illustrates the effectiveness of this workflow for tool development. Using this workflow (Supplemental Fig. 4) makes niche tool development plausible for small working groups that might have less access to the resources needed to produce large batches of annotated data. This pipeline is also faster, cheaper, and more generalizable than immunostaining, which can take days and be prone to investigator bias. This will allow working groups to digitally process many samples within hours instead of spending days immunostaining individual samples.

**Table 3:** Number of training annotations.

	Normal acinar	ADM	Dysplasia
**Number of annotations**			
Image 1	119	1722	1659
Image 2	1342	597	70
Image 3	463	263	3
Total #	1924	2582	1732
**Annotation area (mm**^**2**^**)**			
Image 1	0.05	0.59	1.22
Image 2	0.70	0.17	0.12
Image 3	0.10	0.12	0.02
Total area	0.85	0.88	1.36

There have been many efforts to recreate advanced staining images using more common input modalities^15–18^, and although they are useful for visualizing potential stain and intensity distributions, the algorithms are limited to predicting staining patterns of existing markers. If the user wants to analyze specific biological features for which there is no specific stain; however, simple stain translation will not suffice. The tool created here, however, can create objective binary interpretations of H&E images that segment histologic features of developing pancreatic cancer for which there is no reliable conventional immunostain. Although this methodology uses a conventional UNet architecture^19^, we present a novel and useful application of this technology for studies of pancreatic pre-cancers, classifying and distinguishing the histological features of ADM and PanIN lesions, which have seen few applications before and are important for quantifying pancreatic disease progression. These features, as previously described, are not easily distinguished by any other methods besides manual annotation. Previous studies have attempted to use computer-aided analyses for duct detection in pancreatic cancer^25^, and although the results are good, they are limited in their scope and do not cover a range of subtly different features or early disease hallmarks such as ADM and dysplasia. This illustrates the capacity for modern deep learning methods to provide a broader range of information and perform more complex tasks with comparable accuracy.

Although this tool enables easy, rapid, and accurate binary stain prediction and feature labeling in the early stage disease models employed here, there are several limitations to its predictive capacity. The most prominent source of error for the tool currently is the way it handles unlearned tissue types, such as lymph nodes, pancreatic islets, the desmoplastic stroma, and the occasional presence of neighboring gastrointestinal tissue. Lymph nodes and gastrointestinal tissue are highly irregular compared to the pancreatic features that were present in the training data, leading to completely arbitrary labeling of the unrecognized tissue areas. To overcome this, these regions can simply be cropped prior to analysis, as performed for our analyses. Islets comprise a small fraction of the pancreatic tissue area, and were labeled by the model as “other” (i.e. neither normal, ADM, or dysplasia), and therefore introduced only minor errors. In addition, the desmoplastic stroma is a prominent and histologically distinct feature of pancreatic disease that is currently unlearned and labeled as "other" tissue.

Greater limitations arise with the appearance of high-grade neoplasia and adenocarcinoma, both of which can adopt ductal or disorganized structures more closely resembling ADM. It should also be noted that the tool currently labels all non-neoplastic ductal structures as ADM, whether they originate from acinar cells or from ductal cells, and this contributes some error for the quantification ADM of acinar origin. At this stage of the tool’s development, no label for fully developed adenocarcinoma features were used, so lesions that have progressed beyond high grade dysplasia would likely be mislabeled as either ADM or “other”. With future work, it should be possible to train models to identify these additional tissue features and predict them accurately alongside the existing models. The final limitation of the tools is its failure to make accurate predictions in areas of tissue folding or out of focus imaging, but these are obstacles for any image-based measurement tool (including human annotators) and are avoidable with good technique.

Further work is in progress to reduce error and allow for a broader range of tissue interrogations, including training the tool to recognize a greater diversity of cell types and tissue features such as islets of Langerhans, neural tissue, desmoplastic stroma, adenocarcinoma, and peripheral elements such as lymph nodes or gastrointestinal tissue. The model’s quantitative capabilities can also be applied to other disease states that share similar histologic features, such as pancreatitis. Continued development can yield a single comprehensive tool for predicting and labeling all histologic features in pancreatic tissue without the need for complex staining.

Despite the current limitations discussed above, the tool developed here demonstrates clear advantages and superiority to immunostaining for disease quantification in pancreatic pre-cancers. By relying on H&E staining alone, the data acquisition is not only faster and cheaper, but less vulnerable to variable and uneven staining across tissue sections. This consistency and stability of H&E staining eliminates a primary source of error and bias in feature quantification because of manual adjustments needed to threshold immunostained tissues; tissue immunostaining quality varies significantly within single tissue sections and among the many tissues acquired and stained from animal cohorts, typically stained on different days, months, and even years. This tool’s exploitation of H&E staining not only enables easy quantitative comparisons between tissues collected and stained across broad time periods, but also enables such comparisons among tissues collected and stained in different laboratories around the world. This unifying aspect will improve collaboration and cross-validation between experiments conducted by different groups.

Being computer driven, the tool easily quantifies whole pancreatic tissue sections, allowing greater volumes of data acquisitions and avoiding the selection of “representative” regions for quantification, which introduces further bias. Furthermore, as an automated, machine-driven measurement tool, potential investigator bias is excluded from the data quantification pipeline. Finally, and importantly, tool has been demonstrated to identify and segregate key histologic features which immunostaining methods cannot reliably distinguish (i.e. ADM and dysplasias), significantly extending the power of available tissue analytics. This genre of tool will certainly enhance, and conceivably fully replace immunostaining in many animal studies.

## Methods

### Dataset

Murine pancreatic tissues displaying a range of pre-cancerous lesions were isolated from the *P48*^+*/Cre*^*; LSL-KRAS*^*G12D*^ mice (KC) mouse pancreatic cancer model. This a widely used genetically engineered mouse model of oncogenic Kras-driven pancreatic adenocarcinoma that closely models the evolution of the human disease, displaying the early hallmarks of ADM, Dysplasia, and desmoplasia, and eventually invasive adenocarcinoma after more than one year of age^8^. Tissue sections from 3 whole pancreases were acquired from KC mice at 5 months for models training. This labeled dataset was split into training (80%), validation (20% held out from training), and a first testing dataset (20%). Whole pancreas sections from an additional 25 mice were collected at 2 and 5 months of age (n = 12, n = 13) for IF Spearman correlation testing on a second unseen dataset. Collected pancreases displayed abundant pre-cancerous lesions but were preceding the development of adenocarcinoma. Acute pancreatitis (induced in mice by injection of the pro-inflammatory agent) and normal pancreas sections (n = 6, n = 3) were also collected for generalizability testing on a third unseen dataset. All pancreas tissue sections were stained with H&E and the second testing set was also stained by immunofluorescence for amylase, labeling normal acini, pan-keratin, labeling primarily the oncogenic Kras-transformed epithelial population, and DAPI, labeling all nuclei. These stains were chosen as they are known and well-established markers in the pancreas. Amylase (AMY) is a secretory product of acinar cells, cytokeratins (panK) are well characterized pancreatic ductal lineage markers^26^, and DAPI stains cell nuclei which is used as whole tissue area marker. We use AMY, panK and DAPI combination to identify acinar cells from ADM and PanIN tissues. Acinar cells are positive on AMY but negative for panK; ADM tissues are negative for AMY but positive for panK; PanIN tissues are negative for AMY but positive for panK. Normal acini, ADM, and PanINs have nuclei and can be stained with DAPI.

### H&E staining and immunofluorescence

The pancreatic tissues were paraffin-embedded, sectioned at 5 μm thickness, and H&E stained by standard protocols at the OHSU Histopathology Core. For immunofluorescence staining of amylase and pan-keratin, antigen retrieval was performed using Dako Target Retrieval Solution at pH 9 (Aligent: S236784-2) according to manufacturer’s instructions. Specimens were blocked with blocking buffer (1X PBS/5% normal serum/0.3% Triton X-100) for 1 h at room temperature. The anti-amylase (Santa Cruz: sc-12821) and anti-pan-Cytokeratin (Santa Cruz: sc-15367) primary antibodies were incubated overnight at 4 °C, then washed and incubated with secondary antibodies (Invitrogen: A10042 and A32814) for 1.5 h at room temperature. Slides were covered by coverslips with DAPI's Prolong gold anti-fading agent (Invitrogen: P36931). Fluorescent images of amylase (A), pan-cytokeratin (B), and DAPI (C) staining were acquired using a Carl Zeiss Axioscan Z1 slide scanner at a resolution of 0.2 microns/pixel and converted to BigTiff format.

Immunofluorescence images were quantified using ImageJ software. The threshold tool was applied manually to select the amylase-, pan-cytokeratin, or DAPI-positive tissue regions by trained experts. Lymph nodes were manually cropped and excluded.

Despite all data coming from internal sources, steps were taken to better ensure and test the generalizability of models. Each sample of H&E and IF were collected and stained on different days over the course of several month, and samples were taken at different stages of disease progression. Although H&E samples were stained by the same Histopathology Core, it is likely that staining was done by different operators and used different machines. Following model development, generalizability and robustness to H&E staining differences were tested using synthetically altered H&E stains to show model consistency (Supplemental Fig. 5). Synthetic HE stains were created by randomly shifting the R, G, and B channels by up to ± 25% and applying Gaussian noise. Dice scores were calculated against the unperturbed model predictions. The high mean dice scores support that the model is self-consistent across stains.

### Expert annotation

Annotations for pancreatic tissue features were constructed in Cytomine^20^ by three trained experts, and affirmed by a pathologist. These annotations came from 5 regions across 3 images (Supplemental Fig. 4) and included at total of 1924 normal acinar, 2582 ADMs, and 1732 Dysplasia (Table 3).

### Training image preparation

In order to make the images more amicable to training for the Deep Learning algorithms, they were trained with intensity normalization to make them appear more consistent with each other. To overcome differential staining across an H&E image, various normalization approaches were applied on intermediate sized (5000 × 5000 pixel) overlapping crops prior to tiling (512 × 512 pixel). Background intensities were also ignored from the normalization process to reduce drastic changes on edge regions, isolating only the areas of interest for normalization. Background area was selected by thresholding pixels where all RGB values were greater than 200. The best normalization method was shown to be Reinhard normalization^21^ (Table 2), so it is used in the implementation of the models.

### UNet training

A separate UNet model was trained for each annotated ductal tissue type (normal acinar, ADM, and Dysplasia)^19^. To make each model specific to its respective tissue type, each model’s training set was made to incorporate small portions of the other tissue types as negative controls. The training sets were made using 80% of the total relevant tissue tiles and ~ 5–10% of the total of other tissue tiles. Tiles were augmented during training with flips, rotations, and shears to overcome the small dataset size. Training for all three models lasted for 50 epochs, used a batch size of 32 tiles and had a learning rate of 7e-4, implementing the Adam optimizer. Binary cross entropy (1) was used as the loss function during training. Dice Coefficient (2) was used following training to select the best models.

1 $${\varvec{Binary\, Cross\, Entropy\, Loss}}= -\frac{1}{N}\sum_{i=0}^{N}{y}_{i}\cdot \mathrm{log}\left( \widehat{y}\right)+\left(1-{y}_{i}\right)\cdot \mathrm{log}\left(1-{\widehat{y}}_{i}\right)$$

2 $${\varvec{Dice\, Coefficient}}=\frac{2 \left( X \cap Y \right)}{\left|X\right|+\left|Y\right|}$$

### Model integration

As a standard, models produced through Deep Learning packages will call anything with a prediction value ≥ 0.5 as positive and anything < 0.5 as negative. This threshold, however, might not be the ideal and can be subject to optimization and tuning. Within the training and validation datasets, it was noticed that the standard thresholds led to pixel level false positive noise and predictions that bleed into surrounding ductal lumen. To make the models more accurate, thresholds were chosen based on the Receiver Operating Characteristic (ROC) curves (Supplemental Fig. 6)—sensitivity and specificity, and were manually adjusted to reduce the observed errors qualitatively. This step would help to ensure that the models would better generalize to the testing dataset with minimal noise, taking only predictions the model was most confident in. Within the testing a validation set, the following thresholds were chosen for each model respectively, and the chosen thresholds were carried forward to be used in subsequent testing: Normal Acinar Threshold = 0.3, ADM Threshold = 0.5, and Dysplasia Threshold = 0.7.

After manual parameter tuning, the determined thresholds remain within a reasonable range, as observed by the ROC curves. Once each model made its prediction for a given tissue, the background white pixels were again removed from prediction by ignoring all pixels where all RGB values were greater than 200. Total tissue (DAPI positive) region was also calculated by finding all pixels where RGB values were lower than 200. To combine all four tissue masks, normal acinar predictions override metaplasia and dysplasia predictions; metaplasia predictions override dysplasia predictions; normal acinar, metaplasia, and dysplasia predictions all override DAPI predictions.

### Validation and testing

Because no foreign tissue was used for negative controls during training (primarily lymph nodes and GI tissue), regions of testing images containing these tissues had to be cropped out prior to testing and analysis. Testing and analysis were performed through a similar pipeline as training, incorporating intermediate crop normalization and tile level prediction. These overlapping tiles were stitched back into a full image and an average was taken to get pixel level predictions for each model. In validation, models were compared to annotations from the held-out dataset of labeled images. In testing, model predictions were compared to three unseen datasets: the first comprised of labeled tiles, the second comprised of immunostained serial sections that were thresholded by an expert, and a third comprised of normal and pancreatitis whole tissue sections. To compare with immunostaining, ADM and dysplasia predictions were combined to make a general pan-keratin prediction mask. Predictions were then paired with their respective serial section and correlated to determine model accuracy. Correlation was chosen as the metric for this test over Dice or sensitivity because serial sections have a 5 $\mu m$ offset which causes the H&E used for predictions and the IF used for ground truth to be spatially unaligned. Although correlation of abundances remains high between serial section^24^, the errors in alignment have strong negative biases on metrics like Dice even after attempts at registration. Using IF as ground truth also adds biological credibility to the metrics while annotated ground truths were found to be prone to annotator error.

Like what is done with other virtual staining method that have been deployed on tissue sections^27–29^, we also evaluated our predictions against IF staining was also done with structural similarity^30^:

3 $$\begin{array}{c}SSIM\left(x,y\right)= \frac{\left({2\mu }_{x}{\mu }_{y}\right)\left(2{\sigma }_{xy}+{({k}_{2}L)}^{2}\right)}{\left({\mu }_{x}^{2}+{\mu }_{y}^{2}+{({k}_{1}L)}^{2}\right)\left({\sigma }_{x}^{2}+{\sigma }_{y}^{2}+{({k}_{2}L)}^{2}\right)}\end{array}$$

where x and y are input images, μ_x_ and μ_y_ are the mean intensities, σ_x_^2^ and σ_y_^2^ are the variances, σ_xy_ is the covariance, k_1_ and k_2_ are constants, and L is the dynamic range. SSIM was calculated using the scikit-image library with all default parameters^31^: sliding window size = 7 pixels; k_1_ = 0.01; k_2_ = 0.03; and data range estimated from images. The SSIM between two images is calculated over pixel neighborhoods in the images and provides a more coherent measure of image similarity than pixel-wise measures. We chose SSIM as the metric for comparison because it would be more robust than Dice against differences between the serial H&E and IF sections, and small Gaussian blurs were applied to account for tissue differences at the pixel level. The range of SSIM values extends from − 1 to + 1, and only equals 1 if the two images are identical. Values close to one are indicative of good reconstruction and strong model performance. Four ROIs from two whole slide test sections that had high correspondence between H&E and IF were used for analysis.

The amylase, pan-keratin and DAPI area were measured in pixels, and the percentage of positive areas were calculated as a percent of the total all measured cellular regions.

### Statistical analyses

Since datasets were continuous, independent, and had no tied values, after checking the assumption and conditions were met, and since the datasets were small, non-Gaussian, and contained outliers, the non-parametric Mann–Whitney U test was used to access statistical differences in means. Since datasets were small and had outliers, the correlation tests for all models were conducted using Spearman correlation.

### Animal models

This work was performed in accordance with Institutional Animal Use and Care Committee (IACUC) guidelines of the Oregon Health and Science University (OHSU). All work involving mice received approval by the IACUC at OHSU. The KC mice were all backcrossed at least 5 generations into the C57Bl6/J background. Acute pancreatitis was induced in 6-week old C57Bl6/J mice by intraperitoneal injection of 50 µg caerulein (Sigma:C9026) per kg body weight, with a total of 7 consecutive treatments at 1hour intervals. Pancreatic tissues were harvested 3 days following caerulein treatment. Caerulein was dissolved in PBS at a concentration of 10 µg/ml.

## Supplementary information

Supplementary Information.

## Data Availability

The tool’s code for making predictions is provided on GitHub at the following link: https://github.com/GelatinFrogs/MicePan-Segmentation. Images needed to run the tool can be found in the following google drive: https://drive.google.com/drive/folders/1ipgkjPawkuoLqtLENjHvSVC7hcZKWbRJ?usp=sharing.
