# Supplementary material for: VISTA: VIsual Semantic Tissue Analysis for pancreatic disease quantification in murine cohorts
Source: Sci Rep. 2020 Dec 1;10:20904. doi: 10.1038/s41598-020-78061-3 (PMC7708430; doi:10.1038/s41598-020-78061-3)
Supplement: Supplementary file 1 — Supplementary Information. [file 41598_2020_78061_MOESM1_ESM.pdf]

Title:

VISTA: VIsual Semantic Tissue Analysis for pancreatic disease quantification in murine cohorts

Authors:

Luke Ternes[1,2,#], Ge Huang [1,#], Christian Lanciault[3], Guillaume Thibault[1], Rachelle Riggers[1], Joe W. Gray[1,4], John Muschler[1,5.] *, Young Hwan Chang[1,2,4,5] *

#: equal contribution, *: co-corresponding

Affiliations:

1. Department of Biomedical Engineering and OHSU Center for Spatial Systems Biomedicine (OCSSB)
2. Computational Biology Program, Oregon Health & Science University, Portland, OR
3. Department of Pathology, Oregon Health & Science University, Portland, OR
4. Knight Cancer Institute
5. Brenden-Colson Center for Pancreatic Care

Contact Information:

chanyo@ohsu.edu

All changes to the manuscript are highlighted in yellow with track changes (comment)

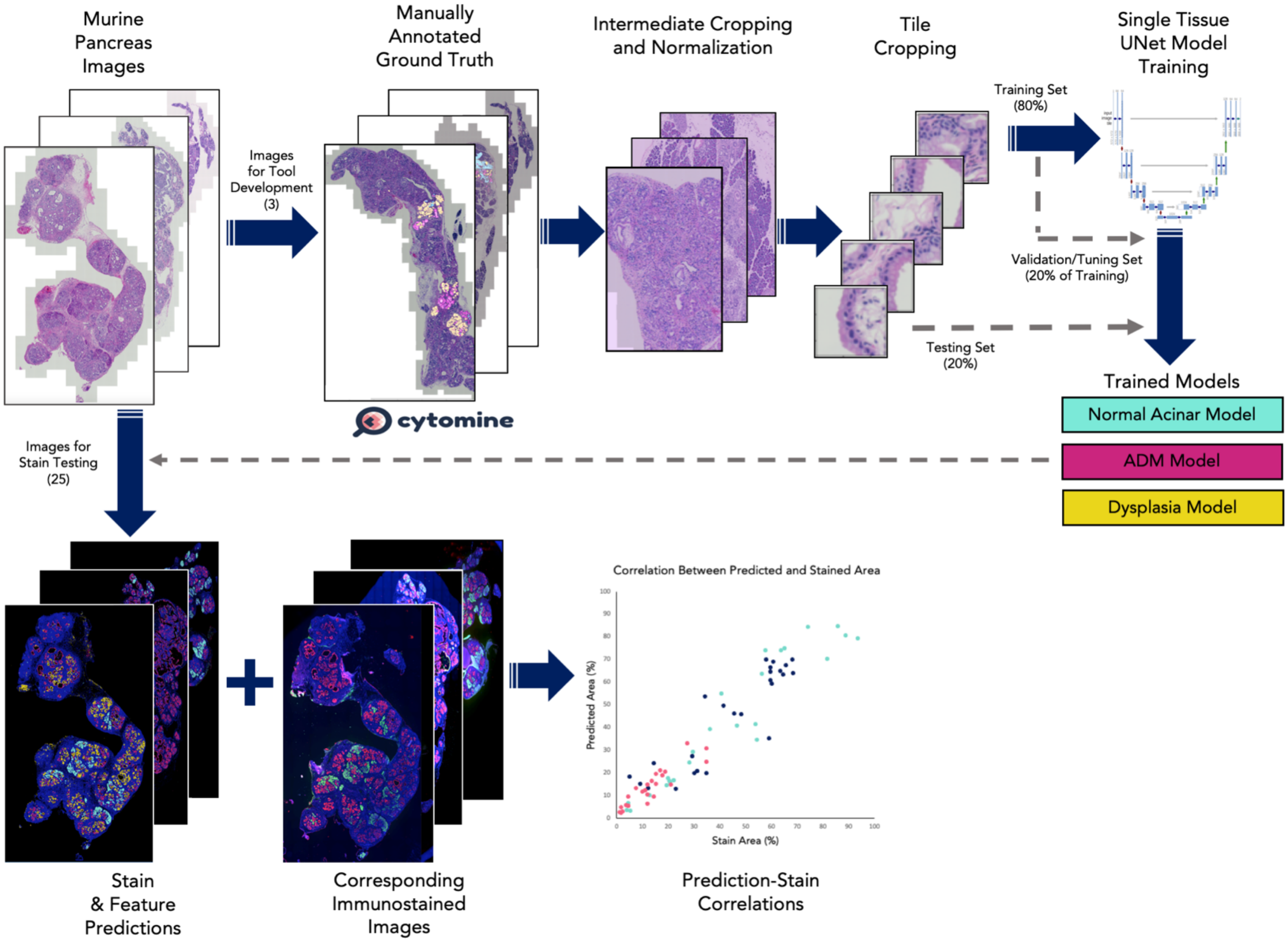

Supplemental Figure 1: Experiment workflow.
A subset of murine pancreas H&E images were annotated by three experts in Cytomine[20]. The images and their annotations were cropped and normalized at intermediate intervals, and these intermediate crops were then tiled into images that can be fed into a UNet architecture[19]. 80% of tiles were used for training and validation, and 20% of tiles were used for testing. A model was trained for each histologic feature label. The best models were chosen and used to predict stain and feature distributions on unseen H&E images. These predictions were then correlated with the stained image counterparts to determine model accuracy.

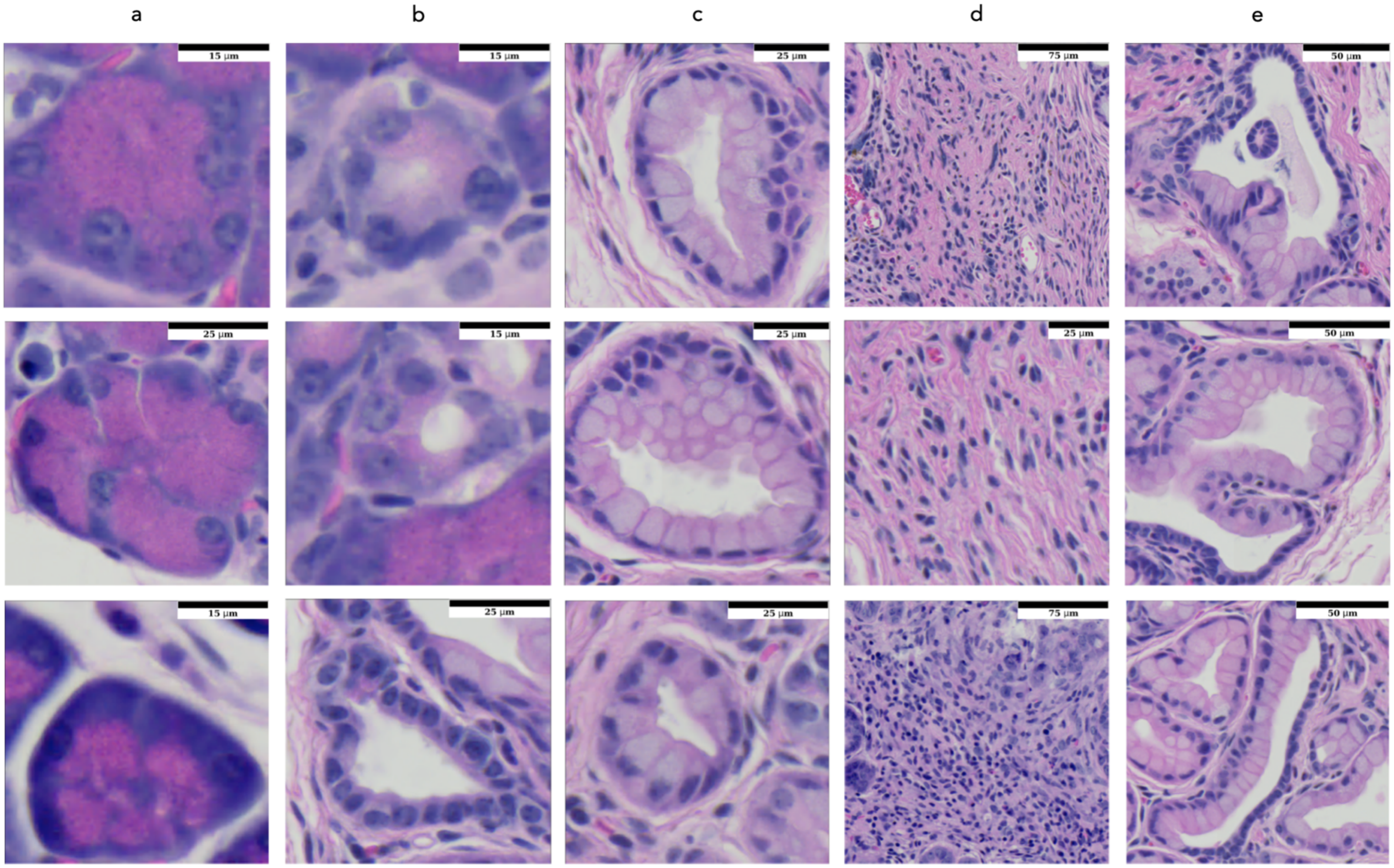

Supplemental Figure 2: Examples of relevant histologic features.

The most prominent histologic features of the pre-cancerous pancreas are the normal acinar epithelial cells (a), the ductal structures resulting primarily from ADM (b), dysplasias (c), and the inflammatory and ECM-rich stroma (d). The normal acini are marked by a thick and darkly stained cytoplasm. ADM is distinguished by a diminished stain, reduced cytoplasm and frequent appearance of a ductal lumen. Low-grade dysplasias are distinguished primarily by an enlargement of the cytoplasm that is lightly stained and correlates with enhanced intracellular mucin production. Dysplasia can exhibit a hybrid appearance of flattened duct-like cells and thickened mucin-rich neoplasia (e). The reactive stroma is marked by dense ECM, spindle like fibroblasts, and inflammatory cells.

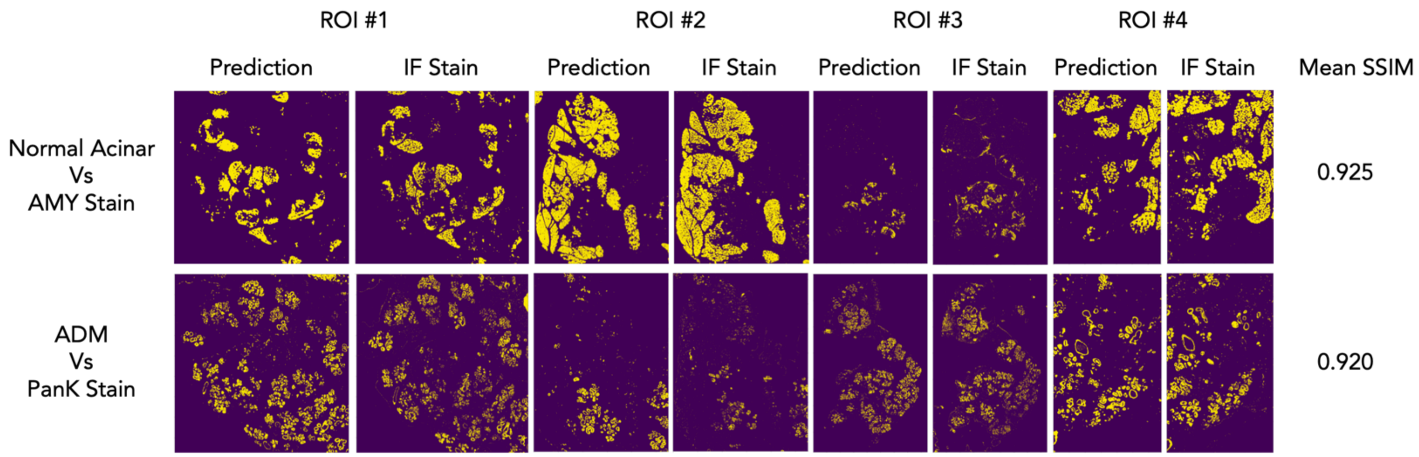

Supplemental Figure 3: Structural similarity index measure between prediction and immunofluorescence. Binarized stain was obtained using thresholding and compared to the corresponding histological predictions for 4 ROIs using SSIM. Small Gaussian blurs were used to account for the fact that samples were taken in serial sections. The high SSIM score support the accuracy of the tool at predicting histological information.

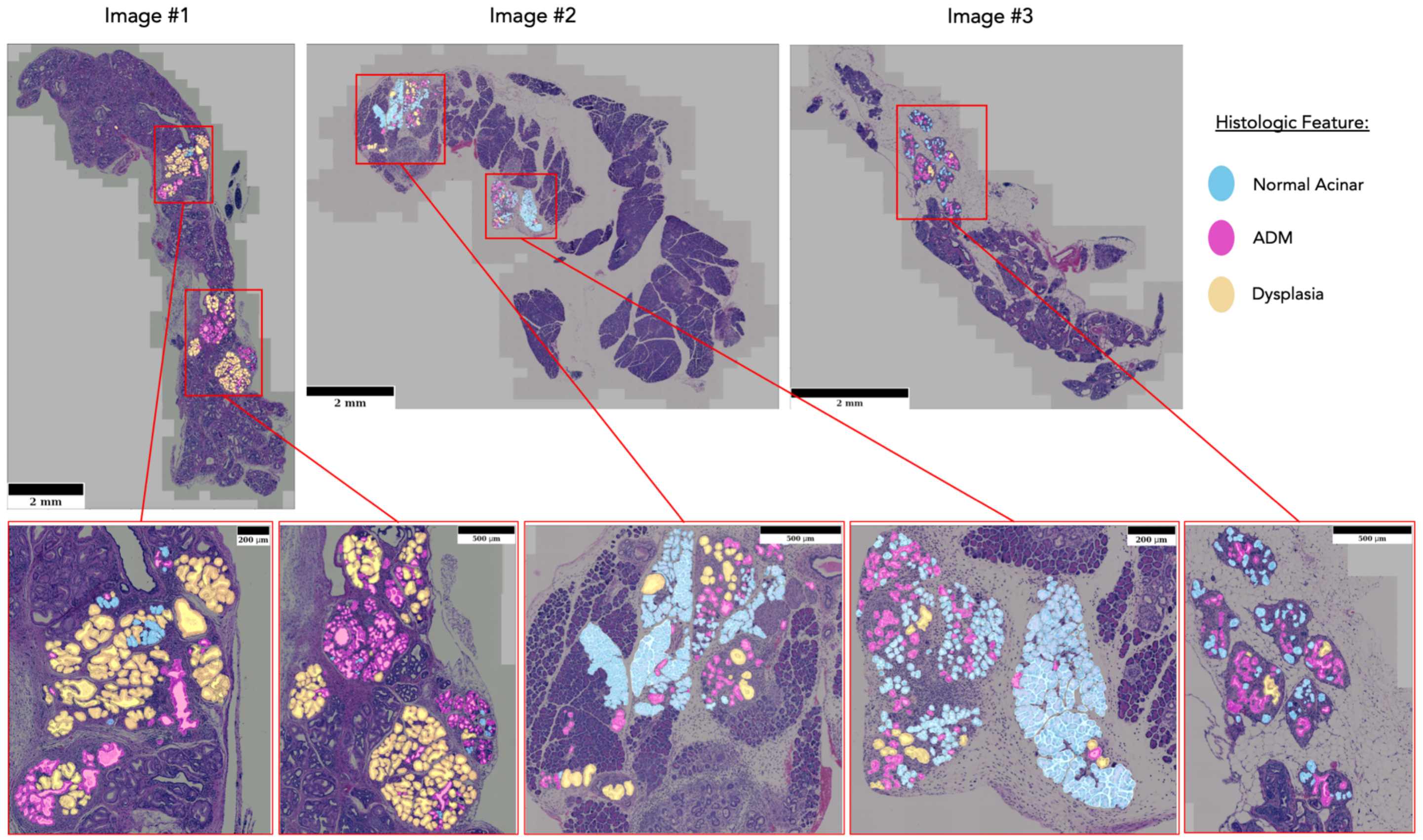

Supplemental Figure 4: Images and regions used for training.
The annotated training, validation, and testing dataset was comprised of labels from 5 regions across three images. In the context of deep learning, where some models are trained on thousands of whole tissue sections, this is considered a small dataset. Despite only being trained on annotations from 3 images, however, the models are successfully generalizable to many more images with different staining qualities and levels of cancer development.

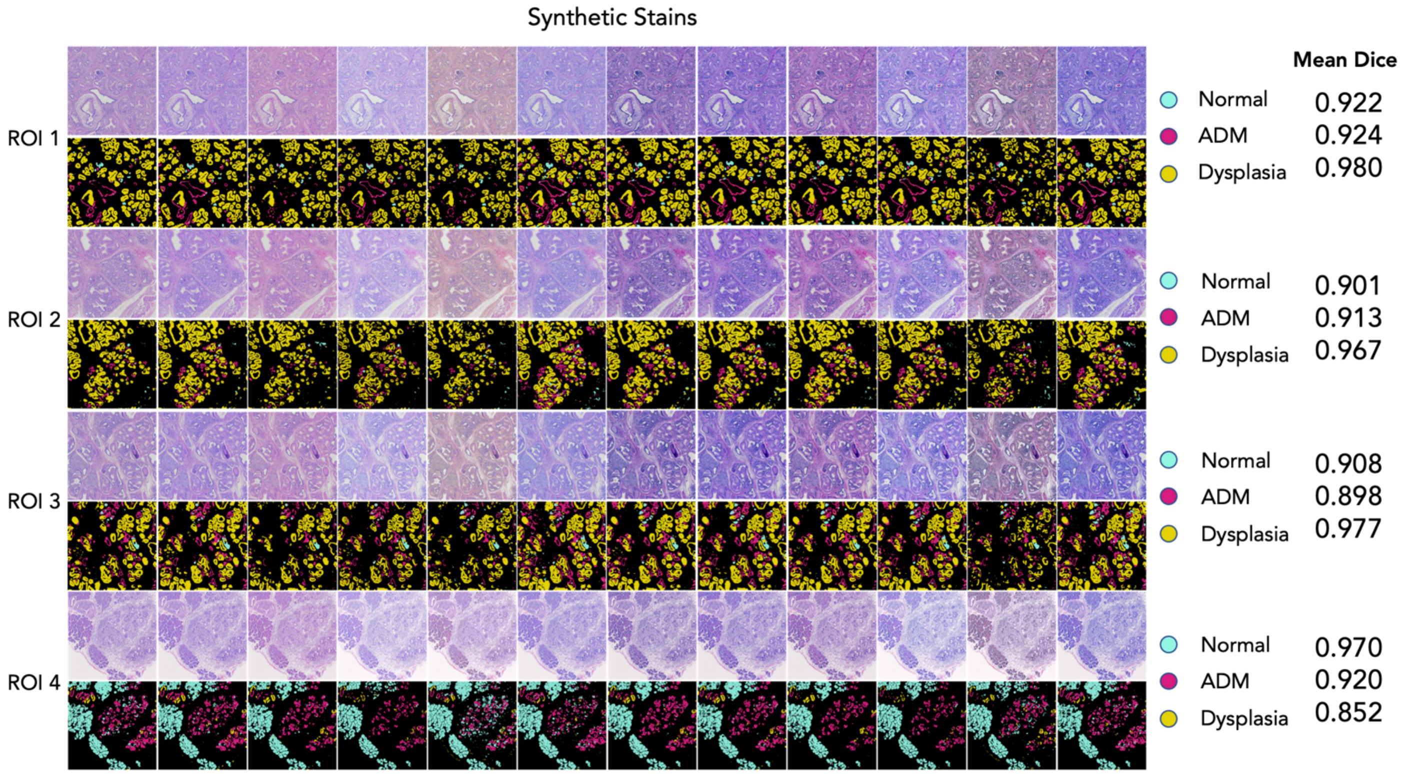

Supplemental Figure 5: Generalization across synthetically generated stains.
Synthetic H&E stains were created by randomly shifting the R, G, and B channels by up to +/- 25% and applying Gaussian noise. Synthetically stained images were then passed through the same prediction pipeline to test model robustness across staining qualities. Dice scores were calculated against the unperturbed model predictions. The high mean dice scores support that the model is self-consistent across stains.

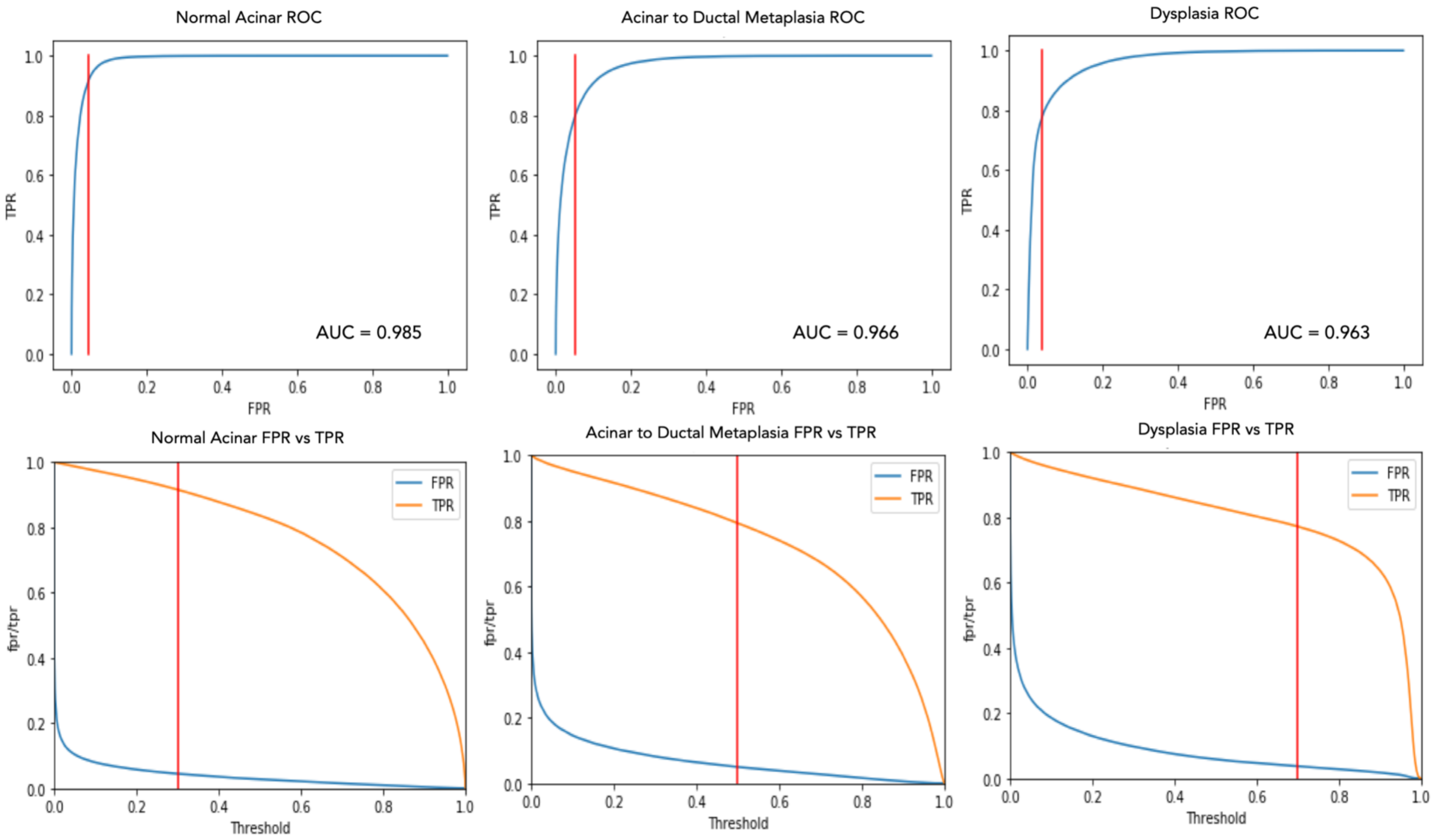

Supplemental Figure 6: ROC curve for models.
ROC curves were created for each model by testing the error rate at 0.1 increments. The red line represents the chosen threshold's corresponding false positive/true positive rates. The high AUC values indicate that the models are highly accurate. The fact that the chosen threshold falls well within the elbow of the ROC curve indicates that despite manual adjustment, the chosen thresholds hold their high predictive value.
